# Child Contact Case Management—A Major Policy-Practice Gap in High-Burden Countries

**DOI:** 10.3390/pathogens11010001

**Published:** 2021-12-21

**Authors:** Anca Vasiliu, Nicole Salazar-Austin, Anete Trajman, Trisasi Lestari, Godwin Mtetwa, Maryline Bonnet, Martina Casenghi

**Affiliations:** 1 TransVIHMI, Institut de Recherche pour le Développement(IRD), Institut National de la Santé et de la Recherche Médicale (INSERM), Montpellier University, 34090 Montpellier, France; maryline.bonnet@ird.fr; 2 Division of Pediatric Infectious Diseases, Department of Pediatrics, School of Medicine, Johns Hopkins University, Baltimore, MD 21287, USA; nsalaza1@jhmi.edu; 3 Internal Medicine Department, Universidade Federal do Rio de Janeiro, Rio de Janeiro 21941-909, Brazil; atrajman@gmail.com; 4 McGill International TB Centre and Department of Medicine, Division of Respiratory Diseases, McGill University, Montreal, QC H3A 0G4, Canada; 5 Center for Tropical Medicine, Universitas Gadjah Mada, Yogyakarta 55281, Indonesia; trisasilestari@gmail.com; 6 Children’s Foundation, Baylor College of Medicine, Houston, TX 77030, USA; gmtetwa@baylorswaziland.org.sz; 7 Department of Innovation and New Technology, Elizabeth Glaser Pediatric AIDS Foundation, 1218 Geneva, Switzerland; mcasenghi@pedaids.org

**Keywords:** tuberculosis, prevention, contact case management, contact investigation, tuberculosis preventive therapy, implementation gaps

## Abstract

The 2021 Global Tuberculosis (TB) report shows slow progress towards closing the pediatric TB detection gap and improving the TB preventive treatment (TPT) coverage among child and adolescent contacts. This review presents the current knowledge around contact case management (CCM) in low-resource settings, with a focus on child contacts, which represents a key priority population for CCM and TPT. Compelling evidence demonstrates that CCM interventions are a key gateway for both TB case finding and identification of those in need of TPT, and their yield and effectiveness should provide a strong rationale for prioritization by national TB programs. A growing body of evidence is now showing that innovative models of care focused on community-based and patient-centered approaches to household contact investigation can help narrow down the CCM implementation gaps that we are currently facing. The availability of shorter and child-friendly TPT regimens for child contacts provide an additional important opportunity to improve TPT acceptability and adherence. Prioritization of TB CCM implementation and adequate resource mobilization by ministries of health, donors and implementing agencies is needed to timely close the gap.

## 1. Introduction

In September 2018, the United Nations (UN) General Assembly called for a united and urgent global response to fight the tuberculosis (TB) epidemic. As part of this call, the following pediatric TB targets were endorsed, with a commitment to achieve them by 2022: diagnose and treat 3.5 million children with TB and 115,000 children with drug-resistant TB; provide TB preventive treatment (TPT) to 4 million contact children under 5 years, 20 million household contacts above 5, and 6 million people living with HIV, including children.

The 2021 Global TB report shows slow progress towards closing the pediatric TB detection gap and meeting the UN targets. Out of the 1.09 million children estimated to fall ill with active TB disease, only 399,000 (36.5%) were notified to national TB programs in 2020, leaving 63.5% undiagnosed or unreported [1]. In the 3-year period 2018–2020, about 1.2 million child contacts under 5 were initiated on TPT, which corresponds to 29% of the 5-year target expected to be achieved by 2022. Progress on contacts aged 5 years and above accessing TPT has been minimal, with only 0.32 million being initiated on TPT between 2018 and 2020, which represents 1.6% of the 5-year target [1].

It is widely accepted that 5–10% of child contacts will develop TB in the first year after exposure [2]. This risk is increased among young children and children living with HIV who are both defined by the World Health Organization (WHO) as high priority groups for contact investigation. Recent evidence shows that 83% of children under 5 years who develop TB, do so within 90 days from their first TB screening visit [2]. In order to prevent progression to TB disease, the WHO recommends screening household and close contacts of all ages shortly after the diagnosis of the index patient and provision of TPT when indicated [3]. Contact case management (CCM) comprises all activities related to the management of contacts of TB patients, from identification, screening, TB investigations, and TPT initiation when indicated.

Implementation of systematic CCM has a critical dual role. First, it allows for timely investigation and diagnosis of TB disease in contacts of all ages, thereby protecting individuals from the development of more severe forms of disease and limiting TB transmission in the community. Second, CCM allows timely provision of TPT to eligible contacts, playing a key role in preventing the onset of TB disease, including in populations at highest risk like children under 5 years and children living with HIV. CCM is therefore a key gateway for both active case-finding and TPT and plays a critical role in breaking the transmission cycle from the index patient to TB-exposed children and to the community. Both at the individual and population level, CCM would thus have an enormous advantage if it was routinely implemented for all TB index patients and their contacts.

## 2. National Policies and the Policy-Practice Gap

At a policy level, the importance of CCM is generally recognized. A recent assessment of national policies related to TB CCM in nine African countries and India, with a primary focus on children and adolescents 0–14 years old, showed that national policies are generally up to date and aligned with international guidance with regard to target populations for CCM and models of care to deploy CCM activities [4]. All ten countries surveyed have national policies that support community-based contact identification and TB screening and allow community health workers (CHWs) to perform those activities. However, expansion of TPT to TB contacts above 5 years of age remains a key challenge. Only half of the countries surveyed reported that child contacts 5–14 years were considered eligible for TPT, irrespective of HIV status.

The evidence that has been generated on the role of contact investigation activities on pediatric TB case finding and in the identification of people who would benefit from TPT is reviewed in Table 1. Systematic reviews that have assessed the prevalence of TB disease among contacts have consistently reported an increased risk of developing active TB disease in child contacts < 5 years of age. In addition, a meta-analysis of individual randomized trials demonstrated that contact investigation increased TB case notification (RR 2.5 [95% CI: 2.0–3.2]), and decreased mortality (RR 0.6 [95% CI: 0.4–0.8]) [5].

Despite supportive evidence and existing WHO and national-level recommendations for CCM, many barriers still exist to their implementation. Recent systematic reviews have identified key challenges for CCM implementation and delivery of TPT, which include knowledge, attitudes, fear of stigma, poor access to tuberculin skin test (TST) and interferon gamma release assay (IGRA) for TB infection identification, poor access to chest radiography (CXR) to rule out active TB disease before TPT initiation, competing priorities for parents, and long treatment regimens affecting treatment adherence [10,11,12]. The impact of interventions deployed to overcome some of these challenges was summarized in an additional systematic review [13]. While thirty-two different interventions were reported to improve different steps of the cascade-of-care for TPT, pooled analysis identified the following key interventions as those having an impact on completion of the TPT cascade of care: patient incentives, healthcare worker education, home visits, digital solutions, and patient reminders [13]. The inclusion of those interventions as part of the standard of care for CCM services could contribute to reducing the observed implementation gaps.

## 3. Key Considerations for Roll-Out of CCM

### 3.1. Target Populations and Screening Approaches

The WHO has recommended contact investigation as a key strategy for TB control since 2012 [14] and has recently updated recommendations for TPT management in close contacts of TB index patients. In 2006, TPT was recommended for child contacts under 5 years of age and for people living with HIV (PLHIV). These populations have been prioritized for TPT delivery because they have a higher risk of progression to TB disease once infected and have a higher risk of severe and disseminated TB. Until recently, CCM of child contacts aged 5–15 primarily aimed at early detection of TB disease. TPT recommendations have progressively evolved to target contacts of all ages, regardless of HIV status. The WHO 2020 guidelines and the accompanying operational handbook recommend that a negative symptom-based TB screen can be considered sufficient to exclude TB disease in child contacts under 5 years, especially in contexts with limited access to CXR, common in many high TB incidence countries [3,15,16,17]. The most recent WHO guidelines on TB screening recommend using symptom screening that includes cough, fever, and poor weight gain (or weight loss) [18]. Similarly, in a pragmatic approach to ensure all at-risk child contacts have access to TPT, the WHO does not require systematic TST or IGRA testing prior to TPT initiation, weighing the low risk of treatment against the relatively high risk of progressing to TB disease. However, contacts over 5 years of age without HIV infection should ideally go through a more intense diagnostic work-up which includes symptom screening, TST or IGRA testing for asymptomatic children to support the identification of people with TB infection, and CXR for those with a positive test result for TB infection, with the aim of ruling out active TB disease. While this more intense diagnostic work-up has the scope to prioritize provision of TPT to the subpopulation that may be in higher need (e.g., those with confirmed TB infection), availability and accessibility to testing for TB infection can be challenging in many TB endemic settings. Tests for TB infection that are currently available on the market are characterized by several limitations, especially for implementation in TB endemic settings [19]. TST has been in use for many years as it is an affordable, simple test, characterized by minimal infrastructure requirements. However, it has significant disadvantages including a second visit after 48–72 h, cold-chain requirements, short supply of quality-assured product, operator-dependent interpretation of results, false positives in individuals vaccinated with Bacillus Calmette–Guérin (BCG), false negatives in cases of immunosuppression, and cross-reactivity with non-tuberculous mycobacteria (NTM). IGRAs offer the advantages of instrument-based result interpretation and improved specificity. Key disadvantages include high cost and significant laboratory infrastructure requirements. Therefore, choice of screening algorithms for older children and adolescents deserves careful consideration by national TB programs based on the availability and accessibility of currently available CXR technologies and tests for infection. While efforts and resources are critically needed to progressively improve access to those technologies, their benefit and added value in different subpopulations need to be considered based on age and risk for progression to TB disease [20]. In settings where CXR, TST or IGRAs are not available, their unavailability should not become a barrier to scaling up TPT and symptom screening should be used for ruling out TB disease in child contacts. 

While current CXR technologies and assays for TB infection have considerable limitations when it comes to implementation in resource limited settings, innovative solutions such as digital and portable CXR, used in combination with computer aided CXR interpretation (CAD) have the potential to improve access to CXR [21,22,23,24,25]. CAD has been recommended by the WHO as an alternative to human reader interpretation of CXR screening for TB in people aged 15 years and above [18]. However, it has not been adequately assessed in children and more data are needed to validate the performance in this population. New versions of TST and IGRA are expected to become available in the near future, all using recombinant ESAT6 and CFP10 antigens. The new skin tests should be characterized by improved accuracy and easier interpretation of results while simultaneously being less costly and requiring minimal laboratory infrastructure. Next generation IGRAs represent simplified versions of the assays that should be suitable for implementation in decentralized facilities [19,26]. These new versions of TST and IGRAs are currently undergoing WHO policy review. It is critical to ensure that these new imaging technologies and TB infection assays are evaluated timely in pediatric populations and more evidence is generated on optimal strategies and algorithmic approaches to exclude TB disease and identify eligible contacts for TPT initiation across the age spectrum. 

### 3.2. TPT Regimens and Availability of Child Friendly Formulations

Short-course TPT regimens hold great promise to improve the safety and completion of TB prevention regimens. The WHO and Center for Disease Control (CDC) have both endorsed short-course TPT regimens including three months of daily rifampicin and isoniazid (3RH), twelve weekly doses of rifapentine and isoniazid (3HP), and four months of daily rifampicin (4R) [3,27]. Additionally, the WHO had endorsed 1 month of daily rifapentine and isoniazid (1HP) in adolescents 13 years and older [3]. These regimens have all been shown to be safe and non-inferior to six to nine months of isoniazid (6H or 9H) in children, with higher completion rates (Table 2) [28,29,30,31,32,33,34,35].

TPT implementation has been hindered by concerns of hepatotoxicity and the development of drug resistance [36]. Nevertheless, children tolerate TPT well and the risk of hepatotoxicity is < 1% for all short course regimens. Further, anticipatory guidance around early signs of hepatotoxicity with early discontinuation of TPT can prevent long-term consequences. Drug resistance can develop when TB disease is not adequately ruled out and one drug therapy is started in the setting of TB disease. While confirming TB diagnosis can be challenging in children, the symptom-based screening approach has good negative predictive value [16] and the risk of developing a new drug resistance mutation in children is less likely given the paucibacillary nature of pediatric TB disease.

Caregivers identified palatability, as well as number and size of tablets as the most important TPT attributes [37]. Additionally, clinical trials and programmatic studies have shown shorter course TPT regimens are associated with higher completion rates [29,32,35]. For young children aged < 5 years, 3RH is preferable given the dispersible fixed dose combination tablet, low daily pill burden, and ease of procurement because it is already being used in many settings for the continuation phase of TB treatment. Even still, caregivers report pragmatic challenges including difficulty dissolving the tablet and a significant amount of time required to administer it, both of which have hindered families’ ability to integrate TPT administration into their daily routines [38].

The lack of child-friendly formulations has hindered implementation of both the 4R and 3HP regimens. While rifampicin can be made into a suspension, this can often not be accomplished at decentralized sites where TPT is dispensed, thereby limiting the 4R regimen for young children in many settings. 3HP is rapidly being adopted for TB prevention to treat people living with HIV in many LMIC. Its implementation is limited by not only the lack of a child-friendly formulation but also the lack of evidence for safety and dosing for children aged less than 2 years. Pharmacokinetics and safety studies are underway to evaluate a water-dispersible rifapentine tablet, including among those less than 2 years old, but significant delays are expected for its commercial availability due to the identification of a nitrosamine impurity in rifapentine. Experience from the field shows us families not only want child-friendly formulations, but also daily regimens to assist with adherence and fixed dose combinations to reduce pill burden [39]. To facilitate bringing a dispersible rifapentine tablet to market and allowing for more flexibility in the composition of child-friendly rifapentine based regimen, a pediatric fixed-dose combination of rifapentine and isoniazid has not been recommended [40].

Finally, 1HP is an attractive ultra-short course TPT regimen with daily dosing that may enhance adherence compared with 3HP. IMPAACT 2024 will evaluate dosing of 1HP using the film-coated rifapentine tablet in children with and without HIV aged 2 to 12 years. This ultra-short regimen holds great promise once pediatric dosing is known and water-dispersible rifapentine tablets are available.

### 3.3. Models of Care for CCM

Historically, TB control strategies have focused on diagnosis and treatment of active TB disease, with an emphasis on the most infectious form of the disease in order to prevent transmission. With the endorsement of the WHO End TB strategy to control tuberculosis, perspectives have widened, and increasing attention has been reserved to TPT as well as to pediatric TB management [41,42]. CCM has been delivered passively at health facilities for decades.

In the facility-based model, the TB index patients are requested to bring their household and close child contacts to the facility for TB screening and TB investigations. However, several challenges and limitations have been documented when implementing this approach. From a patient or caregiver perspective, bringing the child contacts to the facility for TB screening implies transportations costs and possible loss of income due to the time needed for the medical visits. In most of the cases the child contacts are healthy and therefore the motivation to overcome those hurdles remains low. Long waiting time at the facility and fear of exposing healthy children to potential infections can also explain parents’ reluctance to bring their children [43,44,45]. From a health care provider perspective, overcrowded services and weak prioritization of CCM-related activities often do not leave sufficient time to adequately inform the TB index patient about the importance of CCM [43,46,47]. All these reasons have been amplified by the COVID-19 pandemic with limited transport and increased cost, overburdened facilities and increased fear from parents [48]. The 21% reduction in people receiving TPT [1] hints at the potential impact on CCM of the pandemic and its related restriction measures. However, careful adaptation of CCM activities and integration with the COVID response has been highlighted [49]. Possible COVID-19/TB integrations for contact investigation include improved community awareness for the two diseases, screening for COVID-19 while screening for TB, sample collection, and contact investigation for COVID-19 during the TB screening visit [49].

In a global context with more community interventions for TB case management, which are supported by WHO recommendations [50], an answer to some of these barriers has been a hybrid approach that provides contact identification and screening in the community and TB investigations, TPT initiation and follow-up at the health facility. Different delivery strategies have been assessed, either using healthcare workers or CHWs. When delivered by CHWs, emphasis is put on their training and supervision [51]. Community-based interventions for contact investigation showed significant increase in the yield of contacts identified and screened per TB case (0.40 vs. 0.20, *p* = 0.08) [52]. Despite studies documenting the favorable outcomes of community-based approaches for contact investigation, implementation remains limited. The cost of community-based contact investigation, including additional efforts required by already stretched healthcare providers, has often been cited as a barrier to its implementation [53]. Emerging evidence is showing that enhanced passive approaches to contact investigation (i.e., health workers make reminder phone calls and follow-up with the family and encourage them to bring household contacts to the facility for screening) and community-based approaches effectively identify additional patients with TB among household contacts at a relatively modest cost [54,55]. However, cost-effectiveness of community-based approaches may vary depending on context [56]. Therefore, additional evidence from different epidemiological and economical contexts is needed

A fully decentralized CCM approach, where contact screening, TPT initiation and follow-up take place at patients’ homes has the potential to eliminate several barriers to accessing care. This approach could be more easily integrated in the ongoing community interventions for treatment refill and adherence monitoring of index cases. Evidence related to the feasibility and effectiveness of this approach is currently limited. A cohort study in the Gambia, initiating and delivering TPT in the household has shown 94.5% of children who initiated TPT went on to complete TPT. With regard to the eligible children, 72% completed 6 months of TPT and 61% completed the TPT with good adherence [57]. Vikela Ekhaya, a community-based TB household contact management program in Eswatini demonstrated high rates of TPT acceptance (98%) and completion (93%) among eligible children [58]. In the CRESPIT randomized controlled trial, conditional cash transfers, community meetings, and household visits increased the likelihood of completing TPT by 60% in Peru [59]. Two cluster randomized controlled trials (RCT) are currently evaluating the impact of a community intervention for contact investigation and TPT management compared to the facility-based approach [60,61].

### 3.4. Patient-Centered CCM Approaches

Recent evidence has also emphasized the importance of a person-centered approach to contact investigation, irrespective of the model of care implemented. Person-centered care includes empathy, respect, engagement, relationship, communication, shared decision making, holistic focus, individualized focus, and coordinated care [62]. Showing empathy and respect is crucial in building rapport with the index patient during the initial visit. Different and flexible person-centered approaches for each CCM cascade step may be needed depending on the child or family circumstances [63]. A study in Cali, Colombia, showed that good communication with the index patient can facilitate CCM because the patient is willing to share contact information [64]. Attendance rate at health facilities for TB screening visits was higher for household contacts who received a home visit by health-care workers, compared to schools or workplace contacts, who were only receiving a notification by mail [65]. Involving caregivers in treatment decision making and supporting their preferences emerged as prominent elements of person-centered care in a CCM study in Lesotho [37]. Comprehensive health education to household contacts in Indonesia has increased household contact participation in tuberculosis screening by 1.83- fold and case findings by 3.13-fold [66]. Additional examples of tailored and patient-centered approaches are provided by the model set by HIV with differentiated service delivery approaches [67]. By learning from the HIV experience and adapting it to TB management needs and priorities, acceptable and adaptable options for TPT and counselling services could be made available for child and adolescent contacts eligible for TPT. For example, contact children could be referred to a health center near their school where they could access TPT services, including monthly visits, pill refills, and counselling for adherence. Despite recommendations to implement person-centeredness in CCM, particularly in primary healthcare, the literature describing comprehensive person-centeredness is still limited.

### 3.5. Going beyond Household CCM

As household exposure accounts only for 10–30% of TB transmission as shown by a recent review [68], the scope of CCM should be expanded beyond household contact screening in health facilities or the community, by looking into larger communities, schools, workplaces, and other settings where transmission could potentially occur. In order to reach the population at risk of getting TB after exposure, a comprehensive approach should be used, including a range of community-based strategies with targeted screening activities in other dwellings, in addition to household CCM.

Although young children with TB disease are considered at low risk of transmission given the paucibacillary nature of their disease, older children and adolescents have potential to transmit the infection, as they usually present with adult-like disease. Transmission in school settings has already been documented [69], making schools an important target for CCM [70]. In addition, CCM could identify adults with TB disease in school settings who were the index case for the investigated child [71]. Close collaboration with school health care programs should be ensured for CCM activities, to avoid panic or stigma.

### 3.6. Reporting

Recording tools and close monitoring and evaluation should be ensured for CCM activities. In addition to the yearly reporting to the WHO, there should be constant reporting to the national TB Programs and supervision for CCM reporting activities. CCM recording tools should be longitudinal, starting from the index patient, in order for all steps of both the TB case finding and TPT cascade-of-care to be recorded and followed, from contact identification and screening to identification of presumptive TB and TB diagnosis for symptomatic children and TPT initiation and completion for child contacts identified as eligible. Accurate recording is critical to monitor activities, identify gaps in the cascade of care, and implement adequate corrective actions and solutions.

### 3.7. Key Knowledge Gaps and Future Research

A growing body of evidence is being generated around CCM and shows that at every step of the cascade of care there are specific implementation gaps which can be addressed by tailored solutions as shown in Table 3. Research studies designed to tackle these specific gaps are also suggested.

## 4. Conclusions

In June 2021, the WHO released the Call to Action 2.0 that calls on government and stakeholders to accelerate the coverage of TPT for those in need and highlights the critical gap that we are currently facing in reaching the contacts [72]. The significant improvement achieved in recent years in the TPT coverage among PLHIV [1,20] clearly shows that commitment and prioritization of TB activities by bilateral donors, jointly with increased political will and engagement by national programs, can make rapid progress feasible. This review presents the current knowledge around CCM in low-resource settings, with a focus on child contacts, which represents a key priority population for CCM and TPT. Compelling evidence has been generated on the yield and effectiveness of CCM interventions and this should provide strong rationale for prioritization by national TB programs. A growing body of evidence is now showing that the CCM implementation gaps that we are currently facing can be narrowed by innovative models of care focused on community-based and more patient-centered approaches to household contact investigation. The availability of shorter and child-friendly TPT regimens for child contacts provide an additional important opportunity to improve TPT acceptability and adherence. Prioritization of TB CCM implementation and adequate resource mobilization by ministries of health, donors, and implementing agencies is urgently needed to timely close the gap.

## Figures and Tables

**Table 1:** Review of evidence on yield of prevalent TB disease and TB infection among contacts.

Author	Type of Review	Population	Main Findings (Low- and Middle-income Countries)					
			% of TB Disease among All Age Contacts	% of TB Disease among Children < 5 Years	% of TB Disease among Children 5–14 Years	% of TB Infection among All Ages Contacts	% of TB Infection among Children < 5 Years	% of TB Infection among Children 5–14 Years
Morrison et. al. 2008 [6]	Systematic review and meta-analysis	Household contacts of people with active pulmonary TB	4.5% (95% CI: 4.3–4.8, I² = 95.5%)	8.5% (95% CI: 7.4–9.7%, I² = 88.8%)	6.0% (95% CI: 4.7–7.5%, I² = 43.5%)	51.4% (95% CI: 50.6–52.2, I² = 99.4%)	30.4% (95% CI: 28.6–32.3%, I² = 94.4%)	47.9% (95% CI: 45.5–50.4%, I² = 96.0%)
Fox et al. 2013 [7]	Systematic review and meta-analysis	Contacts of patients with new or recurrent TB	3.1% (95% CI: 2.2–4.4; I^2^ = 99.4%)	10% (95% CI: 5.0–18.9; I^2^ = 97.8%)	8.4% (95% CI: 2.8–22.6; I^2^= 92.5%)	51.5% (95% CI: 47.1–55.8; I^2^ = 98.9%)	35.5% (95% CI: 30.3–41.1; I^2^ = 96.6%)	53.1% (95% CI: 42.0–63.9; I^2^ = 98.6%)
Blok et al 2015 [8]	Comparative meta-analysis	Contacts of patients with smear positive or smear negative TB and EPTB	1.8% (95% CI: 1.2–2.7; I^2^ = 97.8%)	Not available	Not available	Not available	Not available	Not available
Velleca et al 2021 [9]	Systematic review and meta-analysis	Close contacts of patients diagnosed with pulmonary TB and EPTB	2.87% (95% CI: 2.61–3.14, I^2^ = 97.79%)	6.84% (95% CI: 5.56–8.11, I^2^ = 95.95%)	3.13% (95% CI: 2.11–4.16; I^2^ = 85.81%)	43.83% (95% CI: 38.11–49.55, I^2^ = 99.36%)	Not available	Not available
Velen et al 2021 [5]	Systematic review and meta-analysis	Contacts of patients with new or recurrent TB	3.6% (95% CI: 3.3–4.0%; I^2^ = 98.9%)	3.9% (95% CI: 2.5–5.4%, I^2^ = 97.0%)	2.4% (95% CI: 1.6–3.4%, I^2^ = 84.5%)	42.4% (95% CI: 38.5–46.4%; I^2^=99.8%)	37.1% (95% CI: 25.9–48.9%, I^2^ = 97.7%)	50.2% (95% CI: 42.6–57.8%, I^2^ = 95.5%)

CI = confidence interval; EPTB = Extra-pulmonary tuberculosis; TB = tuberculosis.

**Table 2:** Advantages and disadvantages of commonly available TPT regimens in children in low-resource settings.

TPT Regimen	Target Population (Children and Adolescents)	Advantages	Disadvantages
1HP	Children 13 years and above	▪ High completion rates	▪ Not available for children under 13 years
3RH	Children with and without HIV from birth to 18 years	▪ Child friendly formulation for children weighing < 25 kg ▪ High completion rates ▪ Widely available in low-resource settings (used for continuation phase of TB treatment)	▪ No child friendly formulation for children > 25 kg ▪ Drug-drug interactions with OCP, LPV/r and NVP (prophylaxis and treatment)
3HP	Children 2 to 18 years with and without HIV	▪ High completion rates ▪ Low rates of adverse events including hepatotoxicity	▪ No dosing for children aged < 2 years ▪ No child-friendly formulation ▪ Relatively high cost of the regimen ▪ Drug–drug interactions with OCP, LPV/r and NVP (prophylaxis and treatment)
4R	Children with and without HIV from birth to 18 years	▪ High completion rates ▪ Low rates of adverse events including hepatotoxicity	▪ No child friendly formulation
6H	Children with and without HIV from birth to 18 years	▪ Low cost ▪ Few drug-drug interactions ▪ Long experience with this drug ▪ Dispersible tablet available	▪ Long duration, poor completion rates ▪ Relatively more side effects including hepatotoxicity (~1%)

**Table 3:** Review of key implementation gaps and possible solutions.

Steps	Implementation Gaps	Proposed Solutions	Research Needs
Contact identification and screening	Index cases not coming back to the health facility with household members	▪ Community-based model of care ▪ Person-lefted care	▪ Cluster RCTs comparing community-based model vs facility-based model ▪ Qualitative research assessing approaches to community sensitization and patient education/ on the importance of CCM and TPT counselling
	Missed contacts	▪ CCM in schools, workplaces, in addition to the household ▪ Person-lefted care	▪ Operational research to identify the best approaches and strategies for CCM in school settings and workplaces
	Healthcare workers’ sensitization and empowerment	▪ Training health providers on the importance of CCM and on communication skills.	▪ Qualitative research on training needs and sensitization strategies
	Improve quality and fidelity of TB screening for identification of presumptive TB and exclusion of active TB disease	▪ Availability of a biomarker- or biosignature-based triage test for systematic TB screening to identify people with presumptive TB and to rule out active TB disease [58]. ▪ Software for computer aided detection of TB-related abnormalities on chest radiography to be validated in the pediatric population ▪ Introduction and roll-out of portable and digital devices for CXR	▪ Studies aimed at identifying and validating TB biomarkers or biosignatures need to include the pediatric population from the onset ▪ Evaluation of the CAD software in children in particular those below 5 years of age ▪ Operational research studies to evaluate the effectiveness and cost-effectiveness of inclusion of digital CXR and CAD in the TB screening algorithms for children and adolescents.
TB investigations for symptomatic contacts	Limited capacity and confidence by frontline HCWs to clinically diagnose pediatric TB	▪ Integrated TB treatment decision trees for children	▪ Operational research studies validating the TB treatment decision algorithms in different settings and for different subpopulations at risk (i.e., CLHIV, malnourished children)
Diagnosis of TB infection in child contacts above 5 years	Availability of better tools for TB infection investigation (more suitable for implementation at decentralized level and not requiring multiple visits)	▪ Differentiated approaches to testing for TB infection for children, adolescents and adults based on risk assessment ▪ Roll-out of RDT based IGRAs or specific skin tests (undergoing WHO policy review in November 2021)	▪ Operational research studies evaluating the placement of new tests in the diagnostic algorithms for investigation of TB infection and their use and added value in children 5–9 years old and adolescents
TPT initiation	Families not bringing children to the health facility for initiation	▪ Community-based model of care ▪ Person-lefted care	▪ Cluster RCTs comparing community-based model vs facility-based model for initiation of TPT
	TPT for MDR-TB contacts	▪ Roll-out of TPT regimen for child contacts of MDR-TB index cases	▪ Implementation studies to assess optimal assessment and TPT delivery of TPT to MDR-TB exposed children.
TPT follow-up and completion	Lack of adherence Assessing and reporting TPT side effects	▪ Roll-out of available shorter and child friendly regimens ▪ Training healthcare workers on TPT side effects and their management	▪ Pharmacokinetics and safety study of 1HP in children, including assessment of drug-drug interactions with commonly used pediatric medicines. ▪ Bioequivalence studies to assess dosing of dispersible rifapentine formulations in children. ▪ Qualitative studies to assess palatability and acceptability of new regimens.
	Contact not returning for follow-up visits	▪ Community-based model of care	▪ Cluster RCT assessing community based TPT management and having as endpoint TPT completion

CAD = computer aided detection; CCM = contact case management; CXR = chest radiography; IGRA = Interferon Gamma Release Assay; MDR = multi-drug resistance; RCT = randomized clinical trial; TB = tuberculosis; TPT = tuberculosis preventive treatment.

## Data Availability

Not applicable.
